# The Effects of Sleep on Emotional Target Detection Performance: A Novel iPad-Based Pediatric Game

**DOI:** 10.3389/fpsyg.2018.00241

**Published:** 2018-03-07

**Authors:** Annalisa Colonna, Anna B. Smith, Stuart Smith, Kirandeep VanDenEshof, Jane Orgill, Paul Gringras, Deb K. Pal

**Affiliations:** ^1^Department of Basic and Clinical Neuroscience, Institute of Psychiatry, Psychology and Neuroscience, King’s College London, London, United Kingdom; ^2^Evelina London Children’s Hospital, Children’s Sleep Medicine, St. Thomas Hospital, London, United Kingdom

**Keywords:** sleep, emotional processing, executive function, polysomnography, children, spindles

## Abstract

**Background:** Consolidation of learning occurs during sleep but when it is disturbed there may be an adverse impact upon these functions. While research has focused upon how sleep affects cognition in adulthood, the effects of disrupted sleep are likely to impact more heavily on learning among children and adolescents. We aimed to investigate whether a night’s sleep impacts upon executive function compared with an equivalent wakefulness period. We also wanted to know whether restricting sleep would reduce these effects on performance. To investigate this issue in children, we adapted existing research methods to make them more suitable for this population.

**Methods:** Using a cross-over trial design, 22 children aged 7–14 completed an updated but previously validated, continuous performance task (CPT) designed to be appealing to children, containing emotional and neutral targets and presented on an iPad. We measured omission and commission errors, mean and variability of reaction times (RTs) immediately and after a delay spent in the following three ways: 11-h intervals of unrestricted and restricted sleep in the style of a ‘sleepover’ and daytime wakefulness. We examined differences in immediate and delayed testing for each dependent variable. Both sleep nights were spent in a specialist sleep lab where polysomnography data were recorded.

**Results:** While there were no significant main effects of sleep condition, as expected we observed significantly faster and more accurate performance in delayed compared with immediate testing across all conditions for omission errors, RT and variability of RT. Importantly, we saw a significant interaction for commission errors to emotional targets (*p* = 0.034): while they were comparable across all conditions during immediate testing, for delayed testing there were significantly more errors after wakefulness compared with unrestricted sleep (*p* = 0.019) and at a trend level for restricted sleep (*p* = 0.063). Performance improvement after restricted sleep was inversely correlated with sleep opportunity time (*p* = 0.03), total sleep time (*p* = 0.01) and total non-REM time (*p* = 0.005).

**Conclusion:** This tool, designed to be simple to use and appealing to children, revealed a preserving effect of typical and disrupted sleep periods on performance during an emotionally themed target detection task compared with an equivalent wakefulness period.

## Introduction

Research with healthy children and adults shows specific memory consolidation occurring during sleep (Marshall and Born, 2007; Wilhelm et al., 2008). Poor sleep quality and quantity can adversely affect memory and cognition including spatial learning (Nguyen et al., 2013), language consolidation (Tamminen et al., 2013) as well as executive function (EF). Much of this research has involved adult participants, although the impact of poor sleep is likely to be even more pronounced during childhood and adolescence (Anderson, 2002).

The challenge for pediatric research is that many of the carefully controlled conditions, measures and paradigms used in adult research are not likely to suit or appeal to children. Furthermore there is evidence that information presented for overnight learning paradigms may need to be particularly salient and engaging for memory consolidation in young people (Stickgold and Walker, 2013).

The development of accessible child-focused measures designed to be sensitive to overnight effects of sleep on memory and cognition are crucial to allow larger pediatric studies to quickly quantify the impact of sleep interventions. The development of a set of tasks designed around a ‘gaming’ approach with an animated child narrator to allow salience, engagement, and ease of use has recently been described (Colonna et al., 2015).

Previous work by Soffer-Dudek et al. (2011) showed that children who experience disruptive sleep are more likely to make omission and commission errors on a target detection task. Sadeh’s team employed a reliable and valid task designed to be engaging to children and adolescents (Rosenberg-Kima and Sadeh, 2010). The stimuli consisted of faces embedded within balloons that drifted upward on a computer screen at a range of speeds, some of which were targets and needed to be ‘popped’ using a mouse. Target definition varied across three different conditions determined either by color of the balloon (green), gender of the face (girl), or emotional expression of the face (happy). Intriguingly, Sadeh’s team found sleep disturbances impacted on performance on the emotional processing condition only.

The emotional value of information being processed has been shown to have both a positive and a negative effect upon performance, largely based upon the perceived threat of the emotional stimuli: if the emotional content of the stimulus is non-threatening or ambiguous this may enhance responding to that stimulus to facilitate further investigation but this may well be quite a subtle change and will be less detectable (Pessoa, 2009). However, where stimuli are perceived as threatening, it is thought that the response strategy appears to become more global, possibly drawing on other resources and in this way increasing responses even to non-targets (Verbruggen and De Houwer, 2007). Previous work with adults has shown that disrupted sleep is negatively associated with emotional EF (Sagaspe et al., 2006; Yoo et al., 2007; Killgore et al., 2008; Franzen et al., 2009; van Der Helm et al., 2010). In children, recognition of emotional stimuli increases with sleep (Prehn-Kristensen et al., 2013) while nap withdrawal in toddlers increases the likelihood of negative responses regardless of whether an image is positive or negative and nap withdrawal also leads to negative rather than confused responses to unsolvable puzzles (Berger et al., 2012).

Given that Sadeh’s task had already been shown to be sensitive to sleep disruption in adolescents, it seemed a suitable paradigm to develop further using the programming potential and end-user appeal possible in a contemporary tablet platform. This study set out to repeat and expand the findings of Sadeh’s original study with an updated tool by comparing changes in EF in a wide age range of typically developing children after a period of either unrestricted or restricted sleep with an equivalent daytime period. We tested the hypotheses that (a) performance on the target detection task would improve following unrestricted sleep relative to an equivalent daytime period and (b) restricted sleep would lead to compromised performance change relative to unrestricted sleep.

Previous study designs measuring sleep and cognitive performance in children and adolescents have often been observational, exploring correlations between amount of sleep and performance change, rather than experimental, using control or comparison groups (Astill et al., 2012). Importantly, uncontrolled studies cannot differentiate circadian effects from sleep effects and cannot compare sleep with wakefulness. We chose a balanced crossover trial design to address these issues and allow us to take advantage of also measuring objective standardized sleep parameters.

## Materials and Methods

### Design

This was a balanced crossover trial comparing performance across three types of testing session separated by an interval of at least 1 week: daytime period (D), overnight unrestricted sleep period (S), and an overnight partial restricted sleep period (R). Based on our intended comparison of D with first S and then R, we created five different order groups (SDR; SRD; DSR; RDS; RSD) so there were equal numbers of children with the order DS (*n* = 13) and SD (*n* = 14), and also DR (*n* = 11) and RD (*n* = 12).

### Participants

We recruited a convenience sample of 30 typically developing children (10 female, 20 males) aged 7–14 years old (mean: 11.05 ± 3.45). All children spoke English as their first language, had normal hearing and vision, no medical or psychiatric disorders likely to affect sleep and none took hypnotic medication.

### Measures

#### Executive Functioning

**FIGURE 1:**
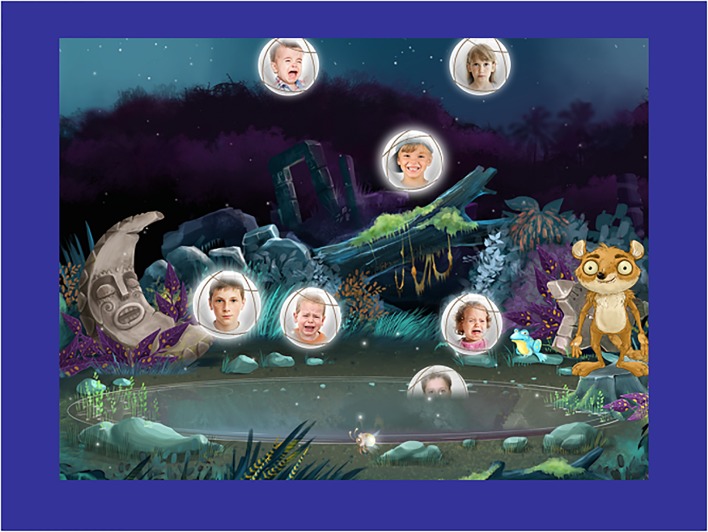
A screen shot of the Pond Poppers task.

Working with Sadeh, we adapted the previously described Balloons Task (Rosenberg-Kima and Sadeh, 2010) into “Pond Poppers,” an animated target detection game, with two conditions requiring either emotional or gender processing. Like the original game, the goal was to burst predefined target bubbles (rather than balloons) amongst non-target bubbles drifting upward on the screen using a fingertip rather than a mouse as in the original (see **Figure 1**). In the emotionally neutral condition, targets were defined by gender (target faces: girls; non-target faces: boys). In the emotional condition, targets were defined by emotion (target faces: happy; non-targets: sad or neutral). For each condition, there were 70 targets and 130 non-targets, the ratio used in the original task (Rosenberg-Kima and Sadeh, 2010). While the original task used three discrete speeds at which the balloon went from the bottom to the top of the screen, Pond Poppers used a smooth gradient instead, beginning with 2.5 s and ending with 1.1 s for the bubble to travel from bottom to top of screen. Mean reaction time (RT), intraindividual coefficient of variation of reaction time (ICV) (intraindividual standard deviation divided by individual mean), omission (missed targets) and commission errors (non-target hits) were automatically computed and stored wirelessly to a database server. This game was optimized for and presented to the participants on an Apple iPad^TM^ tablet (iPad Air 16GB with 9.7′′ Retina display, 64-bit A7 chip, running iOS version 8.1) with Sony MDR over-ear headphones.

### Sleep

The NPSG montage consisted of an electroencephalogram (EEG) comprised of frontal (F3 and F4), central (C3 and C4) and occipital (O1 and O2) leads with auricular reference electrodes, left and right electro-oculographic (EOG) channels, 3 submental electromyographic channels, 2 lead electrocardiography and pulse oximetry (SpO2). Placement of the EEG electrodes was in accordance with the International 10-20 system (Jasper, 1958). All NPSGs were recorded and analyzed using RemLogic software (versions 2.0.2, 3.2 and 3.4), the Embla N7000 recording system and Embla MDrive/Embla communication unit. Nonin xpod cables and disposable oximeter probes were integrated (via the Embla Patient Unit) to measure SpO2 and pulse rate.

Four senior physiologists from the Evelina London Children’s Hospital Children’s Sleep Centre scored the data with repeat scoring by the Chief Physiologist (KvE). The analysis of sleep stages and arousals was performed using 30-s epochs, in accordance with the latest American Association of Sleep Medicine guidelines (Berry et al., 2012). Sleep measures (measured in minutes) included:

• Sleep Opportunity Time
• Total Sleep Time (TST) (the total amount of time the patient is actually asleep (so in stage one, two, three, and REM sleep) thus not including wake periods)
• Amount of REM (Rapid eye movement) as percentage of TST
• Amount of non-REM sleep as percentage of TST
• Amount of REM as percentage of sleep period (the time elapsed from the first epoch of sleep to the final epoch of sleep thus including awake periods)
• Amount of non-REM as percentage of sleep period
• Amount of Total REM
• Amount of Total non-REM

Electroencephalogram rhythm physiological variables were also collected and will be reported in a separate article.

### Cognition and Behavior

Parents completed the Children’s Sleep Habits Questionnaire (CSHQ) (Owens et al., 2000) and Strengths and Difficulties Questionnaires, SDQ (Goodman, 2001). A cognitive neuropsychologist (ABS) and neurodevelopmental pediatrician (AC) administered a brief IQ-screening assessment, including the Wechsler Abbreviated Scale of Intelligence (Wechsler, 1999) and Digit forward and backward subtests (Test Of Memory and Learning, 2nd Ed.) (Reynolds and Voress, 2007).

### Ethics Statement

The study was approved by King’s College London College Research Ethics Committee. Adult studies have employed complete overnight sleep deprivation but this is not ethically acceptable for children and young people. We were given ethical permission for mild sleep restriction, to mimic the effects of a ‘sleepover’ that adolescents typically experience. Before participation, parents and children gave written informed consent and assent.

### Procedures

Both sleep conditions (unrestricted and restricted) took place in the Sleep Disorders Centre at Guy’s Hospital, London, with 5–8 participants taking part in each session.

For both sleep nights, immediate testing on the task took place on Saturdays at 7.30 pm after preparation for NPSG recording. The next morning subjects were woken 11 h after testing (between 6.30 and 7.00 am) and delayed testing took place 60 min later, to avoid any effects of sleep inertia on performance (Wertz et al., 2006). During unrestricted sleep, participants went to bed with lights out at a time determined as typical for this set of children and for typical UK children and in keeping with their usual bedtime (between 9.00 and 10.00 pm) (Blair et al., 2012). During restricted sleep, participants underwent moderate sleep restriction by watching movies, eating pizza and going to bed at 1.00 am, with lights out between 1.30 and 2.00 am. All research procedures were supervised by members of the research team.

The daytime condition took place in a quiet room at each participant’s home at home on Saturdays with immediate testing in the morning and delayed testing in the evening after an 11-h wake period. For all conditions, participants were asked to avoid caffeine and any stressful or cognitively demanding activities that might interfere with learning.

### Data Analysis

Data were analyzed using Statistical Package for Social Sciences (SPSS) version 21.0.

To compare the effects of time of day on performance, we used a one-way ANOVA to compare baseline scores during immediate testing only for all three conditions.

To examine order effects that may have occurred after each testing session, we used a one-way within-subjects ANOVA and repeated contrasts to test all six testing occasions for order effects.

To explore effects of each condition, a two-way within-subjects ANOVA was carried out with sleep condition (daytime, unrestricted, and restricted sleep) and testing time (immediate and delayed) as within-subject variables for each dependent variable.

We used Pearson’s correlations to explore associations between significant performance changes during both sleep nights and neuropsychological and PSG measures.

## Results

### Missing Data Analysis

**FIGURE 2:**
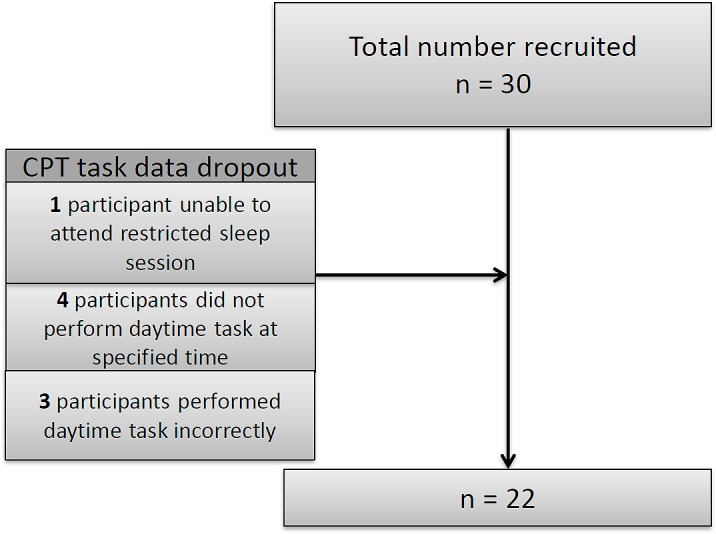
Flow diagram of recruitment process.

Thirty children were recruited for the study but there were some drop-outs (see **Figure 2**): one child did not attend the restricted night; four children did not conform to daytime protocol and three children completed the task incorrectly. Since these children were excluded from the analysis we compared them with those remaining in the study in terms of age, gender and ability and found no significant differences.

### Time of Day

We compared immediate testing performance only across all three sleep conditions to examine whether performance differed depending on the time of day. There was no significant effect of time of day for any of the dependent variables.

### Order Effects

There were no order effects for commission errors to emotional (*F*_5,105_ = 1.2; *p* = 0.31) or gender targets (*F*_5,105_ = 0.88; *p* = 0.42).

Significant main effects of order were observed for mean RT to gender targets (*F*_5,105_ = 35; *p* < 0.0001) and emotional targets (*F*_5,105_ = 23; *p* < 0.0001), ICV for gender targets (*F*_5,105_ = 4.9; *p* = 0.002) and emotional targets (*F*_5,105_ = 2.4; *p* = 0.041); omission errors to gender targets (*F*_5,105_ = 26; *p* < 0.0001) and emotional targets (*F*_5,105_ = 38; *p* < 0.0001). Details of *post hoc* tests are given in **Table 1** and described below.

**Table 1:** Means and Standard deviations for each dependent variable for first, second and third session (order effects).

	First session		Second session		Third session	
Variable	Test 1 Immediate	Test 2 Delayed	Test 3 Immediate	Test 4 Delayed	Test 5 Immediate	Test 6 Delayed



**RT (secs)**	**Mean (*SD*)**					
Gender	1.51 (0.19)	1.34 (0.16)	1.28 (0.17)	1.27 (0.12)	1.24 (0.12)	1.21 (0.13)
Emotion	1.67 (0.14)	1.54 (0.15)	1.48 (0.17)	1.43 (0.13)	1.44 (0.16)	1.38 (0.15)



**ICV (secs)**	**Mean (*SD*)**					
Gender	0.36 (0.07)	0.31 (0.07)	0.30 (0.07)	0.31 (0.04)	0.29 (0.04)	0.29 (0.07)
Emotion	0.41 (0.07)	0.38 (0.07)	0.36 (0.06)	0.36 (0.06)	0.38 (0.06)	0.37 (0.07)



**Omission errors (number)**	**Mean (*SD*)**					
Gender	20.14 (8.0)	15.18 (6.7)	12.36 (6.9)	11.18 (7.0)	9.27 (5.4)	8.73 (4.9)
Emotion	34.36 (7.6)	25.77 (8.5)	25.50 (8.5)	20.50 (7.4)	20.05 (7.3)	18.82 (8.1)



**Commission errors (number)**	**Mean (*SD*)**					
Gender	6.73 (6.0)	5.86 (7.7)	4.32 (4.0)	9.32 (16.9)	6.41 (5.4)	6.14 (5.6)
Emotion	12.27 (9.8)	13.36 (11.1)	11.64 (7.4)	16.64 (14.5)	16.68 (12.6)	13.91 (8.9)

*For all dependent variables Session 1 < Session 2; Session 2 < Session 3 (p < 0.01) except ICV errors Session 2 vs. Session 3 and commission errors for all sessions.*

### Reaction Time Speed

*Post hoc* tests for the gender condition showed that children were faster the second time compared to the first, (*F*_1,21_ = 44; *p* < 0.0001) and again from second to third time (*F*_1,21_ = 4.8; *p* < 0.04). During the emotional condition children became faster across the six sessions: they were significantly faster the second time compared to the first (*F*_1,21_ = 21; *p* < 0.0001) and from second to third time, third to fourth and fifth to sixth time (*F*_1,21_ = 4.0; *p* = 0.058; *F*_1,21_ = 3.7; *p* = 0.068; *F*_1,21_ = 4.5; *p* = 0.045) but with no change from fourth to fifth time.

### Reaction Time Variability

Reaction time were significantly less variable to gender targets the second time the participants did the task compared with the first (*F*_1,21_ = 7.3; *p* = 0.013), but variability did not decrease any further. *Post hoc* tests for variability of RTs to emotional targets did not demonstrate any significant differences.

### Omissions

Mean omission errors to gender targets decreased significantly the second time the participants did the task compared with the first (*F*_1,21_ = 22; *p* < 0.0001) and then from second to third time (*F*_1,21_ = 4.8; *p* = 0.04). After this omission errors plateaued. Mean omission errors to emotional targets decreased significantly from first to second time (*F*_1,21_ = 46; *p* < 0.0001) and then from third to fourth time they did the task (*F*_1,21_ = 29; *p* < 0.0001).

### Sleep Effects

#### Main Effect of Condition

There were no significant main effects of sleep condition for either the gender or emotion condition for any of the dependent variables (see **Table 2**).

**Table 2:** Means and standard deviations for each dependent variable for each sleep condition.

	Daytime		Unrestricted sleep		Restricted sleep	
Variable	Immediate	Delayed	Immediate	Delayed	Immediate	Delayed



**RT (secs)**	**Mean (*SD*)**					
Gender *a^∗∗∗^*	1.35 (0.2)	1.26 (0.16)	1.33 (0.2)	1.29 (0.14)	1.35 (0.16)	1.26 (0.1)
Emotion *a^∗∗∗^*	1.5 (0.2)	1.46 (0.2)	1.5 (0.17)	1.46 (0.12)	1.52 (0.18)	1.41 (0.15)



**ICV (secs)**	**Mean (*SD*)**					
Gender	0.33 (0.07)	0.31 (0.06)	0.29 (0.07)	0.29 (0.06)	0.31(0.063)	0.30 (0.059)
Emotion *a^∗∗^ b^∗^*	0.39 (0.06)	0.37 (0.06)	0.36 (0.069)	0.37 (0.071)	0.39 (0.05)	0.35 (0.06)



**Omission errors (number)**	**Mean (*SD*)**					
Gender *a^∗^*	14.09 (8.9)	13.6 (8.7)	13.68 (8.8)	10.31 (6.1)	14.00 (6.9)	11.2 (4.6)
Emotion *a^∗∗∗^*	28.05 (10.4)	22.72 (9.0)	26.04 (9.5)	20.36 (8.3)	25.81 (9.6)	22.00 (8.1)



**Commission errors (number)**	**Mean (*SD*)**					
Gender	5.36 (4.9)	10.45 (17.8)	5.68 (6.3)	4.86 (3.8)	6.41 (4.6)	6.00 (5.6)
Emotion *b^∗∗^*	11.95 (6.4)	19.27 (16.1)	12.59 (9.7)	11.14 (6.9)	15.95 (13.5)	13.50 (8.7)

*a = significant main effect of testing time; b = significant interaction effect of condition and testing time (no main effect of sleep) ^∗^p < 0.10 (trend)*, *^∗∗^p < 0.05*, *^∗∗∗^p < 0.01*.

#### Main Effect of Testing Time

There was a significant effect of testing time for RT to gender targets (*F*_2,42_ = 26; *p* < 0.0001) and emotion targets (*F*_2,42_ = 31; *p* < 0.0001), variability of response to emotional targets only (*F*_2,42_ = 5; *p* = 0.032) and for omission errors to gender targets (*F*_2,42_ = 13; *p* = 0.002) and emotion targets (*F*_2,42_ = 88; *p* < 0.0001). There was no significant main effect of testing time during either gender or emotion condition for commission errors.

#### Interaction Between Condition and Testing Time

During the emotional condition, there was a significant interaction between condition and testing time for commission errors (*F*_2,42_ = 3.6; *p* = 0.046).

**FIGURE 3:**
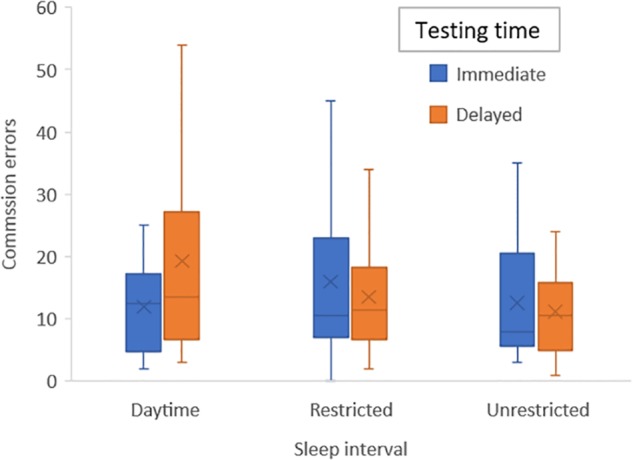
Bar graph of mean number of emotion commission errors for each sleep condition (daytime, unrestricted sleep, and restricted sleep) and for immediate and delayed testing.

To control for learning effects and given that this occurred primarily from first to second time of playing the game we added a variable defining the first session (daytime, sleep or restricted) into our original ANOVA models: The significant interaction for commission errors remained significant (*F*_2,38_ = 3.7; *p* = 0.034) and differences were analyzed with *post hoc* tests to interpret this outcome and to acquire effect sizes: there were no significant differences in number of errors during immediate testing for either condition, but during delayed testing there were significantly more errors during the daytime condition compared with unrestricted sleep (*p* = 0.019; η^2^ = 0.26) and at a trend level for the restricted sleep condition (*p* = 0.063; η^2^ = 0.17) (see **Figure 3**).

### Correlational Analysis

#### Correlations With Change in Commission Errors

Performance change was defined as the difference between immediate to delayed testing expressed as a percentage of number of commission errors at immediate testing (positive value indicates improvement in performance).

During the unrestricted night, we found a positive correlation approaching significance between age and performance change (*r* = 0.42; *n* = 22; *p* = 0.06) so that with increasing age, performance improved from immediate to delayed testing. There was no significant association with sleep quality as measured by the total sleep disturbance as measured by (SHQ).

During the restricted night we observed significant negative correlations with performance change and total sleep opportunity time (*r* = -0.47; *n* = 22; *p* = 0.03), total sleep time (*r* = -0.54; *n* = 22; *p* = 0.01) and total non-REM (-0.58; *n* = 22; *p* = 0.005) so that performance improved with decreased sleep duration (see **Table 3**).

**Table 3:** Means standard deviations (*SD*) for each polysomnography (PSG) measure for each sleep condition and correlations (*r*) between each PSG measure and difference in immediate and delayed commission errors for each condition.

Sleep features	Emotion commission errors performance change							
	Unrestricted sleep				Restricted sleep			
	*n*	Mean (*SD*)	*r*	*p*	*n*	Mean (*SD*)	*R*	*p*
Sleep opportunity (TRT) (mins)	22	519.3 (53.3)	-0.07	0.77	22	317.4 (63.0)	-0.47	0.03
Total sleep time (TST) (mins)	18	446.0 (53.5)	0.04	0.87	22	288.4 (62.3)	-0.54	0.01
% NRM of TST	16	85.1 (3.2)	0.25	0.36	22	88.9 (4.6)	0.14	0.53
% NREM of sleep period	16	75.4 (6.3)	0.13	0.62	22	82.3 (8.6)	-0.23	0.31
% REM of TST	16	14.9 (3.3)	-0.24	0.37	22	10.8 (4.1)	-0.16	0.47
% REM of sleep period	16	13.6 (3.9)	-0.20	0.45	22	10.4 (4.4)	-0.19	0.39
Total NREM (mins)	16	380.8 (49.2)	0.09	0.73	22	244.68 (59.1)	-0.58	0.005
Total REM (mins)	16	66.5 (16.7)	-0.17	0.51	22	33.1 (18.6)	-0.33	0.14

No other significant associations were observed.

## Discussion

In this current study, we have shown that it is possible to preserve the validity of a previously established paradigm (Soffer-Dudek et al., 2011) whilst presenting this to children on a contemporary tablet device and using updated graphics. Importantly, our findings reflect those of the original observational study upon which ours is based. This original study showed that the number of commission errors was positively correlated with overnight disruptions to sleep (Soffer-Dudek et al., 2011), thus demonstrating an association between sleep and commission errors in a very similar task.

We found both restricted and unrestricted sleep appeared to preserve performance, in contrast to an equivalent daytime period after which performance declined. Other studies have shown that sleep can affect other cognitive functions in this way. For example, the performance of adults using motor memory tasks has worsened over a daytime period compared to a night time period (Rickard et al., 2008; Ribeiro Pereira et al., 2015; Nettersheim et al., 2015). Our findings suggest that we can extend this finding to target detection processes in children.

We based our game on a paradigm that had already been shown to be sensitive to sleep in children and adolescents. Relatively little research has been carried out that examines the impact of sleep on EF in children but a recent meta-analysis demonstrated that EF is influenced by sleep restriction in children and is associated with modest effect sizes (Astill et al., 2012). While EF encompasses a wide band of skills, target detection has produced reasonable effect sizes of *d* = 0.5 where targets are defined by their emotional status. Our results confirm that this particular EF is sleep sensitive and suggest that our game has potential for development as an alternative indicator of sleep deficiency.

Sleep during restricted conditions also preserved performance compared with an equivalent daytime period at a level that approached significance. The conditions we provided during our restricted night aimed to replicate ‘sleep-over’ conditions, where children watched a movie and went to bed later than normal. We acquired precise measures of sleep quantity to confirm that sleeping hours and corresponding REM and non-REM time were reduced during this night (see **Table 3**). Thus, our study seems to support other research which shows that children have resilience to acute partial sleep deprivation, compensating EF reasonably well and in fact, we found an inverse correlation between performance and sleep duration and non-REM sleep, which suggests that sleep processes may work in a compensatory way when sleep is undisturbed but restricted. It seems likely that chronic rather than acute sleep deprivation impacts upon performance in children and adolescents as indicated by earlier studies (Anderson et al., 2009; Molfese et al., 2013; Kuula et al., 2015; de Bruin et al., 2016).

The ability to detect emotional information is likely to have particular relevance, since identifying emotional expressions plays a key role in social communication and is related to psychopathology and adjustment in childhood and adolescence when these skills are developing, and in adulthood (Phillips et al., 2003; Leppänen, 2006). Our study supports earlier work showing that emotional intelligence is impacted upon by sleep deprivation (Killgore et al., 2008).

### Limitations

The main limitation of this study is the relatively small group of participants, and different age groups studied. Whilst the ability to capture full sleep, polysomnographic physiological data is important and sets this study apart from many, it also increases the cost and complexity, and arguably the monitoring itself can alter sleep quality. The iPad autonomous platform lends itself to testing large numbers of children remotely (without full polysomnography) and we are also pursuing this alternative model. The hospital setting for overnight sleep interventions, and home setting for daytime conditions may have influenced the children’s task performances, although previous research in a different population of young people found no effect of setting (Galer et al., 2015).

We recognize that although the discriminative task was one of recognizing images of facial emotions, we cannot be certain that this is a task that really taps the processing of emotional expression, as the faces can also be conceptualized as simply more challenging complex images than the gender images. Our future work will focus on equalizing the cognitive load in order to disentangle this confound.

Despite our decision to test children 1 h after waking, we recognize that sleep inertia is a potential confounder and is likely to impact upon performance and some children can take up to 4 h to return to their normal performance. This may have a greater effect after a night of sleep deprivation than a night of normal sleep which may well act as a confounder (Sadeh et al., 2002). However, in this sample of children we didn’t see a difference in commission errors in the morning following restricted compared with non-restricted sleep so sleep inertia seems not to have had a differential effect on this kind of sleep in this sample of children.

## Conclusion

Using a unique gamified tool specifically developed for children, we have shown that sleep has a preserving effect upon a specific facial emotion image recognition task and this skill is conserved even after a single night of restricted sleep.

The task is engaging, does not require administration by trained psychologists, and can be administered remotely across large populations. Our next goals are to observe the effects of chronic sleep deprivation on the same task and define its properties in children with various neurodisabilities.

## Author Contributions

ABS and AC were involved in development of idea, recruitment, research, and writing of manuscript. SS, KVDE, and JO were involved in research and writing of manuscript. PG and DP were involved in development of idea, planning the research, design and writing of manuscript.

## Conflict of Interest Statement

The authors declare that the research was conducted in the absence of any commercial or financial relationships that could be construed as a potential conflict of interest.
